# Universality of Logarithmic Loss in Successive Refinement †

**DOI:** 10.3390/e21020158

**Published:** 2019-02-08

**Authors:** Albert No

**Affiliations:** Department of Electronic and Electrical Engineering, Hongik University, Seoul 04066, Korea; albertno@hongik.ac.kr; Tel.: +82-2-320-1649

**Keywords:** logarithmic loss, rate-distortion, successive refinability

## Abstract

We establish an universal property of logarithmic loss in the successive refinement problem. If the first decoder operates under logarithmic loss, we show that any discrete memoryless source is successively refinable under an arbitrary distortion criterion for the second decoder. Based on this result, we propose a low-complexity lossy compression algorithm for any discrete memoryless source.

## 1. Introduction

In the lossy compression problem, logarithmic loss is a criterion allowing a “soft” reconstruction of the source, a departure from the classical setting of a deterministic reconstruction. In this setting, the reconstruction alphabet is the set of probability distributions over the source alphabet. More precisely, let *x* be the source symbol from the source alphabet X, and q(·) be the reconstruction symbol which is the probability measure on X. Then the logarithmic loss is given by

$$\begin{array}{c} \ell \left(x,q\right)=\log\frac{1}{q(x)}. \end{array}$$

Clearly, if the reconstruction q(·) has a small probability on the true source symbol *x*, the amount of loss will be large.

Although logarithmic loss plays a crucial role in the theory of learning and prediction, relatively little work has been done in the context of lossy compression, notwithstanding the two-encoder multi-terminal source coding problem under logarithmic loss [1,2], or recent work on the single-shot approach to lossy source coding under logarithmic loss [3]. Note that lossy compression under logarithmic loss is closely related to the information bottleneck method [4,5,6]. In this paper, we focus on universal properties of logarithmic loss in the context of successive refinement.

Successive refinement is a network lossy compression problem where one encoder wishes to describe the source to two decoders [7,8]. Instead of having two separate coding schemes, the successive refinement encoder designs a code for the decoder with a weaker link, and sends extra information to the second decoder on top of the message of the first decoder. In general, successive refinement coding cannot do as well as two separate encoding schemes optimized for the respective decoders. However, if we can achieve the point-to-point optimum rates using successive refinement coding, we say the source is successively refinable.

Although necessary and sufficient conditions of successive refinability is known [7,8], proving (or disproving) successive refinability of the source is not a simple task. Equitz and Cover [7] found a discrete source that is not successively refinable using Gerrish problem [9]. Chow and Berger found a continuous source that is not successively refinable using Gaussian mixture [10]. Lastras and Berger showed that all sources are nearly successively refinable [11]. However, still only a few sources are known to be successively refinable. In this paper, we show that any discrete memoryless source is successively refinable as long as the weaker link employs logarithmic loss, regardless of the distortion criterion used for the stronger link.

In the second part of the paper, we show that this result can be useful to design a lossy compression algorithm with low complexity. Recently, the idea of successive refinement is applied to reduce the complexity of point-to-point lossy compression algorithm. Venkataramanan et al. proposed a new lossy compression for Gaussian source where the codewords are linear combination of sub-codewords [12]. No and Weissman also proposed a low-complexity lossy compression algorithm for Gaussian source using extreme value theory [13]. Both algorithms are successively describing source and achieve low complexity. Roughly speaking, successive refinement algorithm provides a smaller size of codebook. For example, the naive random coding scheme has a codebook of size $e^{nR}$ when the blocklength is *n* and the rate is *R*. On the other hand, if we can design a successive refinement scheme with half rate in the weaker link, then the size of codebook is $e^{nR/2}$ each. Thus, the overall codebook size is $2e^{nR/2}$. The above idea can be generalized to successive refinement scheme with *L* decoders [12,14]

The universal property of logarithmic loss in successive refinement implies that, for any point-to-point lossy compression of discrete memoryless source, we can insert a virtual intermediate decoder (weaker link) under logarithmic loss without losing any rates at the actual decoder (stronger link). As we discussed, this property allows us to design a lossy compression algorithm with low-complexity for any discrete source and distortion pair. Note that previous works only focused on specific source and distortion pair such as binary source with Hamming distortion.

The remainder of the paper is organized as follows. In Section 2, we revisit some of the known results pertaining to logarithmic loss. Section 3 is dedicated to successive refinement under logarithmic loss in the weaker link. In Section 4, we propose a low complexity compression scheme that can be applied to any discrete lossy compression problem. Finally, we conclude in Section 5.

*Notation*: $X^{n}$ denotes an *n*-dimensional random vector $\left(X_{1},X_{2},\ldots ,X_{n}\right)$ while $x^{n}$ denotes a specific possible realization of the random vector $X^{n}$. X denotes a support of random variable *X*. Also, *Q* denotes a random probability mass function while *q* denotes a specific probability mass function. We use natural logarithm and nats instead of bits.

## 2. Preliminaries

### 2.1. Successive Refinability

**Figure 1:**
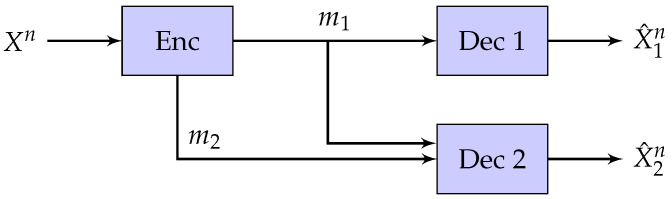
Successive Refinement.

In this section, we review the successive refinement problem with two decoders. Let the source $X^{n}$ be i.i.d. random vector with distribution $p_{X}$. The encoder wants to describe $X^{n}$ to two decoders by sending a pair of messages $\left(m_{1},m_{2}\right)$ where $1\leq m_{i}\leq M_{i}$ for i∈{1,2}. The first decoder reconstructs ${\hat{X}}_{1}^{n}\left(m_{1}\right)\in {\hat{X}}_{1}^{n}$ based only on the first message $m_{1}$. The second decoder reconstructs ${\hat{X}}_{2}^{n}\left(m_{1},m_{2}\right)\in {\hat{X}}_{2}^{n}$ based on both $m_{1}$ and $m_{2}$. The setting is described in Figure 1.

Let $d_{i}\left(\cdot ,\cdot \right):X\times {\hat{X}}_{i}\to \left[0,\infty \right)$ be a distortion measure for *i*-th decoder. The rates of code $\left(R_{1},R_{2}\right)$ are simply defined as

$$\begin{array}{cc} R_{1}= & \frac{1}{n}\log M_{1}\\ R_{2}= & \frac{1}{n}\log M_{1}M_{2}. \end{array}$$

An $\left(n,R_{1},R_{2},D_{1},D_{2},\epsilon \right)$-successive refinement code is a coding scheme with block length *n* and excess distortion probability ϵ where rates are $\left(R_{1},R_{2}\right)$ and target distortions are $\left(D_{1},D_{2}\right)$. Since we have two decoders, the excess distortion probability is defined by $\mathrm{Pr}\left[d_{i}\left(X^{n},{\hat{X}}_{i}^{n}\right)>D_{i}\;\mathrm{for}\;\mathrm{some}\;i\right]$.

**Definition** **1.** *A rate-distortion tuple $\left(R_{1},R_{2},D_{1},D_{2}\right)$ is said to be achievable if there is a family of $(n,R_{1}^{\left(n\right)},R_{2}^{\left(n\right)}$, $D_{1},D_{2},\epsilon^{\left(n\right)})$-successive refinement code where*

$$\begin{array}{c} \lim_{n\to \infty }R_{i}^{\left(n\right)}=R_{i}\;for\;all\;i,\\ \lim_{n\to \infty }\epsilon^{\left(n\right)}=0. \end{array}$$

For some special cases, both decoders can achieve the point-to-point optimum rates simultaneously.

**Definition** **2.** *Let $R_{i}\left(D_{i}\right)$ denote the rate-distortion function of the i-th decoder for i∈{1,2}. If the rate-distortion tuple $\left(R_{1}\left(D_{1}\right),R_{2}\left(D_{2}\right),D_{1},D_{2}\right)$ is achievable, then we say the source is successively refinable at $\left(D_{1},D_{2}\right)$. If the source is successively refinable at $\left(D_{1},D_{2}\right)$ for all $D_{1},D_{2}$, then we say the source is successively refinable.*

The following theorem provides a necessary and sufficient condition of successive refinable sources.

**Theorem** **1** **([7,8]).** *A source is successively refinable at $\left(D_{1},D_{2}\right)$ if and only if there exists a conditional distribution $p_{{\hat{X}}_{1},{\hat{X}}_{2}|X}$ such that $X-{\hat{X}}_{2}-{\hat{X}}_{1}$ forms a Markov chain and*

$$\begin{array}{cc} R_{i}\left(D_{i}\right) & =I(X;{\hat{X}}_{i})\\ E\left[d_{i}\left(X,{\hat{X}}_{i}\right)\right] & \leq D_{i} \end{array}$$

*for i∈{1,2}.*

Note that the above results of successive refinability can easily be generalized to the case of *k* decoders.

### 2.2. Logarithmic Loss

Let X be a set of discrete source symbols (|X|<∞), and M(X) be the set of probability measures on X. Logarithmic loss ℓ:X×M(X)→[0,∞] is defined by

$$\begin{array}{c} \ell \left(x,q\right)=\log\frac{1}{q(x)} \end{array}$$

for x∈X and q∈M(X). Logarithmic loss between *n*-tuples is defined by

$$\begin{array}{c} \ell_{n}\left(x^{n},q^{n}\right)=\frac{1}{n}\sum_{i=1}^{n}\log\frac{1}{q_{i}\left(x_{i}\right)}, \end{array}$$

i.e., the symbol-by-symbol extension of the single letter loss.

Let $X^{n}$ be the discrete memoryless source with distribution $p_{X}$. Consider the lossy compression problem under logarithmic loss where the reconstruction alphabet is M(X). The rate-distortion function is given by

$$\begin{array}{cc} R(D)= & \underset{p_{Q|X}:E\left[\ell (X,Q)\right]\leq D}{\inf}I\left(X;Q\right)\\ = & H(X)-D. \end{array}$$

The following lemma provides a property of the rate-distortion function achieving conditional distribution.

**Lemma** **1.** *The rate-distortion function achieving conditional distribution $p_{Q^{⋆}|X}$ satisfies*

(1) $$\begin{array}{cc} p_{X|Q^{⋆}}\left(\cdot |q\right)= & q \end{array}$$

(2) $$\begin{array}{cc} H(X|Q^{⋆})= & D \end{array}$$

*for $p_{Q^{⋆}}$ almost every q∈M(X). Conversely, if $p_{Q|X}$ satisfies (1) and (2), then it is a rate-distortion function achieving conditional distribution, i.e.,*

$$\begin{array}{cc} I(X;Q)= & R(D)=H(X)-D\\ E\left[\ell (X,Q)\right]= & D. \end{array}$$

The key idea is that we can replace *Q* by $p_{X|Q}\left(\cdot |Q\right)$, and have lower rate and distortion, i.e.,

$$\begin{array}{cc} I(X;Q)\geq & I(X;p_{X|Q}\left(\cdot |Q\right))\\ E\left[\ell (X,Q)\right]\geq & E\left[\ell (X,p_{X|Q}\left(\cdot |Q\right)\right], \end{array}$$

which directly implies (1).

Interestingly, since the rate-distortion function in this case is a straight line, a simple time sharing scheme achieves the optimal rate-distortion trade-off. More precisely, the encoder losslessly compresses only the first $\frac{H(X)-D}{H(X)}$ fraction of the source sequence components. Then, the decoder perfectly recovers those losslessly compressed components and uses $p_{X}$ as its reconstruction for the remaining part. The resulting scheme obviously achieves distortion *D* with rate H(X)−D.

Furthermore, this simple scheme directly implies successive refinability of the source. For $D_{1}>D_{2}$, suppose the encoder losslessly compresses the first $\frac{H\left(X\right)-D_{2}}{H(X)}$ fraction of the source. Then, the first decoder can perfectly reconstruct $\frac{H\left(X\right)-D_{1}}{H(X)}$ fraction of the source with the message of rate $H\left(X\right)-D_{1}$ and distortion $D_{1}$ while the second decoder can achieve distortion $D_{2}$ with rate $H\left(X\right)-D_{2}$. Since both decoders can achieve the best rate-distortion pair, it follows that any discrete memoryless source under logarithmic loss is successively refinable.

We can formally prove successive refinability of discrete memoryless source under logarithmic loss using Theorem 1. I.e., by finding random probability mass functions $Q_{1},Q_{2}\in M\left(X\right)$ that satisfy

(3) $$\begin{array}{cc} I(X;Q_{1})= & H\left(X\right)-D_{1},\;E\left[\ell (X,Q_{1})\right]=D_{1}, \end{array}$$

(4) $$\begin{array}{cc} I(X;Q_{2})= & H\left(X\right)-D_{2},\;E\left[\ell (X,Q_{2})\right]=D_{2}, \end{array}$$

where $X-Q_{2}-Q_{1}$ forms a Markov chain.

Let $e_{x}$ be a deterministic probability mass function (pmf) in M(X) that has a unit mass at *x*. In other words,

$$\begin{array}{c} e_{x}\left(\tilde{x}\right)=\left\{\begin{array}{cc} 1 & \mathrm{if}\;\tilde{x}=x\\ 0 & \mathrm{otherwise}. \end{array}\right. \end{array}$$

Then, consider random pmfs $Q_{1},Q_{2}\in \left\{e_{x}:x\in X\right\}\cup \left\{p_{X}\right\}$. Since the support of $Q_{1}$ and $Q_{2}$ is finite, we can define the following conditional pmfs.

$$\begin{array}{cc} p_{Q_{2}|X}\left(q_{2}|x\right)= & \left\{\begin{array}{cc} \frac{H\left(X\right)-D_{2}}{H(X)} & \mathrm{if}\;q_{2}=e_{x}\\ \frac{D_{2}}{H(X)} & \mathrm{if}\;q_{2}=p_{X}\\ 0 & \mathrm{otherwise} \end{array}\right.\\ p_{Q_{1}|Q_{2}}\left(q_{1}|q_{2}\right)= & \left\{\begin{array}{cc} \frac{H\left(X\right)-D_{1}}{H\left(X\right)-D_{2}} & \mathrm{if}\;q_{1}=q_{2}=e_{x}\mathrm{for}\;\mathrm{some}\;x\\ \frac{D_{1}-D_{2}}{H\left(X\right)-D_{2}} & \mathrm{if}\;q_{1}=p_{X}\;\mathrm{and}\;q_{2}=e_{x}\mathrm{for}\;\mathrm{some}\;x\\ 1 & \mathrm{if}\;q_{1}=q_{2}=p_{X}\\ 0 & \mathrm{otherwise}. \end{array}\right. \end{array}$$

It is not hard to show that the above conditional pmfs satisfies (3) and (4).

## 3. Successive Refinability

### Main Results

Consider the successive refinement problem with a discrete memoryless source as described in Section 2.1. Specifically, we are interested in the case where the first decoder is under logarithmic loss and the second decoder is under some arbitrary distortion measure d(·,·). We only have a following benign assumption that if $d\left(x,{\hat{x}}_{1}\right)=d\left(x,{\hat{x}}_{2}\right)$ for all *x*, then ${\hat{x}}_{1}={\hat{x}}_{2}$. This is not a hard restriction since if ${\hat{x}}_{1}$ and ${\hat{x}}_{2}$ have the same distortion values for all *x*, then there is no reason to have both reconstruction symbols.

The following theorem shows that any discrete memoryless source is successive refinable as long as the weaker link is under logarithmic loss. This implies an universal property of logarithmic loss in the context of successive refinement.

**Theorem** **2.** *Let the source be arbitrary discrete memoryless. Suppose the distortion criterion of the first decoder is logarithmic loss while that of the second decoder is an arbitrary distortion criterion $d:X\times \hat{X}\to \left[0,\infty \right]$. Then the source is successively refinable.*

**Proof.**  The source is successively refinable at $\left(D_{1},D_{2}\right)$ if and only if there exists a $X-\hat{X}-Q$ such that

$$\begin{array}{cc} I(X;Q)= & R_{1}\left(D_{1}\right),\;E\left[\ell (X,Q)\right]\leq D_{1}\\ I(X;\hat{X})= & R_{2}\left(D_{2}\right),\;E\left[d(X,\hat{X})\right]\leq D_{2}. \end{array}$$

Let $p_{{\hat{X}}^{⋆}|X}$ be the conditional distribution for the second decoder that achieves the informational rate-distortion function $R_{2}\left(D_{2}\right)$. i.e.,

$$\begin{array}{c} I\left(X;{\hat{X}}^{⋆}\right)=R_{2}\left(D_{2}\right),\;E\left[d(X,{\hat{X}}^{⋆})\right]=D_{2}. \end{array}$$

Since the weaker link is under logarithmic loss, we have $R_{1}\left(D_{1}\right)\leq R_{2}\left(D_{2}\right)$. This implies that $H\left(X\right)-D_{1}\leq H\left(X\right)-H\left(X|{\hat{X}}^{⋆}\right)$. Thus, we can assume $H\left(X|{\hat{X}}^{⋆}\right)\leq D_{1}$ throughout the proof. For simplicity, we further have a benign assumption that there is no $\hat{x}$ such that $p_{X}\left(x\right)=p_{X|{\hat{X}}^{⋆}}\left(x|\hat{x}\right)$ for all *x*. (See Remark 1 for the case where such $\hat{x}$ exists.)

Without loss of generality, suppose $\hat{X}=\left\{0,1,\ldots ,s-1\right\}$. Consider a random variable Y∈Y={0,1,…,s} with the following pmf for some 0≤ϵ≤1:

$$\begin{array}{c} p_{Y}\left(y\right)=\left\{\begin{array}{cc} \left(1-\epsilon \right)p_{{\hat{X}}^{⋆}}\left(y\right) & \mathrm{if}\;y\leq s-1\\ \epsilon & \mathrm{if}\;y=s. \end{array}\right. \end{array}$$

The conditional distribution is given by

$$\begin{array}{c} p_{{\hat{X}}^{⋆}|Y}\left(\hat{x}|y\right)=\left\{\begin{array}{cc} 1 & \mathrm{if}\;\hat{x}=y\leq s-1\\ 0 & \mathrm{if}\;\hat{x}\neq y\leq s-1\\ p_{{\hat{X}}^{⋆}}\left(\hat{x}\right) & \mathrm{if}\;y=s. \end{array}\right. \end{array}$$

The joint distribution of $X,{\hat{X}}^{⋆},Y$ is given by

$$\begin{array}{c} p_{X,{\hat{X}}^{⋆},Y}\left(x,\hat{x},y\right)=p_{X,{\hat{X}}^{⋆}}\left(x,\hat{x}\right)p_{Y|{\hat{X}}^{⋆}}\left(y|\hat{x}\right). \end{array}$$

It is clear that $H\left(X|Y\right)=H\left(X|{\hat{X}}^{⋆}\right)$ if ϵ=0 and H(X|Y)=H(X) if ϵ=1. Since $H\left(X|{\hat{X}}^{⋆}\right)\leq D_{1}$, there exists an 0≤ϵ≤1 such that $H\left(X|Y\right)=D_{1}$.

We are now ready to define the Markov chain. Let $Q=p_{X|Y}\left(\cdot |Y\right)$ and $q^{\left(y\right)}=p_{X|Y}\left(\cdot |y\right)$ for all y∈Y. The following lemma implies that there is a one-to-one mapping between *q* and *y*.

**Lemma** **2.** *If $p_{X|Y}\left(x|y_{1}\right)=p_{X|Y}\left(x|y_{2}\right)$ for all x∈X, then $y_{1}=y_{2}$.*

The proof of lemma is given in Appendix A. Since $Q=p_{X|Y}\left(\cdot |Y\right)$ is a one-to-one mapping, we have

$$\begin{array}{cc} I(X;Q)= & I\left(X;Y\right)=H\left(X\right)-D_{1}=R_{1}\left(D_{1}\right). \end{array}$$

Also, we have

$$\begin{array}{cc} E\left[\ell (X,Q)\right]= & E\left[\log\frac{1}{p_{X|Y}\left(X|Y\right)}\right]=H\left(X|Y\right)=D_{1}. \end{array}$$

Furthermore, $X-{\hat{X}}^{⋆}-Q$ forms a Markov chain since $X-{\hat{X}}^{⋆}-Y$ forms a Markov chain. This concludes the proof. □

The key idea of the theorem is that (1) is the only loose required condition for the rate-distortion function achieving conditional distribution. Thus, for any distortion criterion in the second stage, we are able to choose an appropriate distribution $p_{X,\hat{X},Q}$ that satisfies both (1) and the condition for successive refinability.

**Remark** **1.** *The assumption $p_{X|{\hat{X}}^{⋆}}\left(\cdot |\hat{x}\right)\neq p_{X}\left(\cdot \right)$ for all $\hat{x}$ is not necessary. Appendix B shows another joint distribution $p_{X,{\hat{X}}^{⋆},Y}$ that satisfies conditions for successive refinability when the above assumption does not hold.*

*The distribution in the above proof is one simple example that has a single parameter ϵ, but we can always find other distributions that satisfy the condition for successive refinability. In the next section, we propose totally different distribution that achieves a Markov chain $X-{\hat{X}}^{⋆}-Y$ with $H\left(X|Y\right)=D_{1}$. This implies that the above proof does not rely on the assumption.*

**Remark** **2.** *In the proof, we used random variable Y to define $Q=p_{X|Y}\left(\cdot |Y\right)$. On the other hand, if the joint distribution $p_{X,{\hat{X}}^{⋆},Q}$ satisfies conditions of successive refinability, there exists a random variable Y where $X-{\hat{X}}^{⋆}-Y$ forms a Markov chain and $Q=p_{X|Y}\left(\cdot |Y\right)$. This is simply because we can set Y=Q, which implies $p_{X|Y}\left(\cdot |Y\right)=p_{X|Q}\left(\cdot |Q\right)=Q$.*

Theorem 2 can be generalized to successive refinement problem with *K* intermediate decoders. Consider random variables $Y_{k}\in Y$ for 1≤k≤K such that $X-{\hat{X}}^{⋆}-Y_{K}-\cdots -Y_{1}$ forms a Markov chain and the joint distribution of $X,{\hat{X}}^{⋆},Y_{1},\ldots ,Y_{K}$ is given by

$$\begin{array}{c} p_{X,{\hat{X}}^{⋆},Y_{1},\ldots ,Y_{K}}\left(x,\hat{x},y_{1},\ldots ,y_{K}\right)\\ =p_{X,{\hat{X}}^{⋆}}\left(x,\hat{x}\right)p_{Y_{1}|{\hat{X}}^{⋆}}\left(y_{1}|\hat{x}\right)\prod_{k=1}^{K-1}p_{Y_{k+1}|Y_{k}}\left(y_{k+1}|y_{k}\right) \end{array}$$

where $H\left(X|Y_{k}\right)=D_{k}$. Similar to the proof of Theorem 2, we can show that $Q_{k}=p_{X|Y_{k}}\left(\cdot |Y_{k}\right)$ for all 1≤k≤K satisfy the condition for successive refinability (where posterior distributions $p_{X|Y_{k}}\left(\cdot |y_{k}\right)$ should be distinct for all $y_{k}\in Y$ to guarantee one-to-one correspondence). Thus, we can conclude that any discrete memoryless source with *K* intermediate decoders is successively refinable as long as all the intermediate decoders are under logarithmic loss.

## 4. Toward Lossy Compression with Low Complexity

As we mentioned in Remark 1, the choice of joint distribution $p_{X,{\hat{X}}^{⋆},Q}$ in the proof of Theorem 2 is not unique. In this section, we propose another joint distribution $p_{X,{\hat{X}}^{⋆},Q}$ that satisfies the conditions for successive refinability. It naturally suggests a new lossy compression algorithm which we will discuss in Section 4.3.

### 4.1. Rate-Distortion Achieving Joint Distribution: Small $D_{1}$

Recall that $H\left(X|{\hat{X}}^{⋆}\right)\leq D_{1}$. We first consider the case where $D_{1}$ is not too large so that $D_{1}$ is close to $H(X|{\hat{X}}^{⋆})$. We will clarify this later. For simplicity, we further assume that $p_{{\hat{X}}^{⋆}}\left(0\right)\geq \cdots \geq p_{{\hat{x}}^{⋆}}\left(s-1\right)$. Consider a random variable $Z_{\epsilon }^{\left(s\right)}\in \hat{X}$ with the following pmf for some $0\leq \epsilon \leq \left(s-1\right)\min_{\hat{x}}p_{{\hat{X}}^{⋆}}\left(\hat{x}\right)$

$$\begin{array}{c} p_{Z_{\epsilon }^{\left(s\right)}}\left(z\right)=\left\{\begin{array}{cc} 1-\epsilon & \mathrm{if}\;z=0\\ \frac{\epsilon }{s-1} & \mathrm{if}\;1\leq z\leq s-1. \end{array}\right. \end{array}$$

If it is clear from context, we simply use $Z\equiv Z_{\epsilon }^{\left(s\right)}$ for the sake of notation. We further define a random variable *Y* that is independent to *Z* such that ${\hat{X}}^{⋆}=Y{⊕}_{s}Z$, where ${⊕}_{s}$ denotes a sum modulo *s*. This can be achieved by following pmf and conditional pmf.

(5) $$\begin{array}{cc} p_{Y}\left(y\right)= & \frac{p_{{\hat{X}}^{⋆}}\left(y\right)-\frac{\epsilon }{s-1}}{1-\frac{s}{s-1}\epsilon }\\ p_{{\hat{X}}^{⋆}|Y}\left(\hat{x}|y\right)= & \left\{\begin{array}{cc} 1-\epsilon & \mathrm{if}\;\hat{x}=y\\ \frac{\epsilon }{s-1} & \mathrm{if}\;\hat{x}\neq y. \end{array}\right. \end{array}$$

If ϵ=0, we have $H\left(X|Y\right)=H\left(X|{\hat{X}}^{⋆}\right)$. Also, it is clear that H(X|Y) increases as ϵ increases. Since we assume that $D_{1}$ is not too large, there exists $0\leq \epsilon \leq \left(s-1\right)\min p_{{\hat{X}}^{⋆}}\left(\hat{x}\right)$ such that $H\left(X|Y\right)=D_{1}$. We will discuss about the case of general $D_{1}$ in Section 4.2. The joint distribution of $X,{\hat{X}}^{⋆},Y$ is given by

$$\begin{array}{c} p_{X,{\hat{X}}^{⋆},Y}\left(x,\hat{x},y\right)=p_{X,{\hat{X}}^{⋆}}\left(x,\hat{x}\right)p_{Y|{\hat{X}}^{⋆}}\left(y|\hat{x}\right). \end{array}$$

We are now ready to define the Markov chain. Let $Q=p_{X|Y}\left(\cdot |Y\right)$ and $q^{\left(y\right)}=p_{X|Y}\left(\cdot |y\right)$ for all y∈Y where $Y=\hat{X}=\left\{0,1,\ldots s-1\right\}$. For simplicity, we assume that $p_{X|Y}\left(\cdot |y_{1}\right)$ and $p_{X|Y}\left(\cdot |y_{2}\right)$ are not the same for all $y_{1}\neq y_{2}$. Since $Q=p_{X|Y}\left(\cdot |Y\right)$ is a one-to-one mapping, we have

$$\begin{array}{cc} I(X;Q)= & I\left(X;Y\right)=H\left(X\right)-D_{1}=R_{1}\left(D_{1}\right). \end{array}$$

Also, we have

$$\begin{array}{cc} E\left[\ell (X,Q)\right]= & E\left[\log\frac{1}{p_{X|Y}\left(X|Y\right)}\right]=H\left(X|Y\right)=D_{1}. \end{array}$$

Furthermore, $X-{\hat{X}}^{⋆}-Q$ forms a Markov chain since $X-{\hat{X}}^{⋆}-Y$ forms a Markov chain. Thus, the above construction of joint distribution $p_{X,{\hat{X}}^{⋆},Q}$ satisfies the conditions for successive refinability.

### 4.2. Rate-Distortion Achieving Joint Distribution: General $D_{1}$

The joint distribution in the previous section only works for small $D_{1}$. It is because ϵ has a natural upper-bound from (5) which is $\epsilon \leq \left(s-1\right)\min p_{{\hat{X}}^{⋆}}\left(\hat{x}\right)$. In this section, we generalize the proof in the previous section for general $D_{1}$. The key observation is that if we pick the maximum $\epsilon =\left(s-1\right)\min p_{{\hat{X}}^{⋆}}\left(\hat{x}\right)$, then $p_{Y}\left(s-1\right)=0$. This implies that we can focus on the smaller set of reconstruction alphabet Y={0,1,…s−2}.

Let $U_{s}={\hat{X}}^{⋆}$, and define random variables $\left\{U_{k}:1\leq k\leq s-1\right\}$ recursively. More precisely, we define the random variable $U_{k-1}$ based on $U_{k}$ for 2≤k≤s.

$$\begin{array}{cc} U_{k}= & U_{k-1}{⊕}_{k}Z_{\epsilon_{k}}^{\left(k\right)}\\ p_{Z_{\epsilon_{k}}^{\left(k\right)}}\left(z\right)= & \left\{\begin{array}{cc} 1-\epsilon_{k} & \mathrm{if}\;z=0\\ \frac{\epsilon_{k}}{k-1} & \mathrm{if}\;1\leq z\leq k-1 \end{array}\right. \end{array}$$

where

$$\begin{array}{c} \epsilon_{k}=\left(k-1\right)\min_{u}p_{U_{k}}\left(u\right). \end{array}$$

Similar to the definition of *Y*, we assume $U_{k-1}$ and $Z_{\epsilon_{k}}^{\left(k\right)}$ are independent, and ${⊕}_{k}$ denotes modulo *k* sum. Each time step, the alphabet size of $U_{k}$ decreases by one. Thus, we have $0\leq U_{k}\leq k-1$, and therefore $U_{1}=0$ with probability 1. Furthermore, we have

$$\begin{array}{c} H\left(X|U_{s}\right)\leq H\left(X|U_{s-1}\right)\leq \cdots \leq H\left(X|U_{1}\right)=H\left(X\right). \end{array}$$

For $H\left(X|{\hat{X}}^{⋆}\right)\leq D_{1}<H\left(x\right)$, there exists *k* such that $H\left(X|U_{k}\right)>D_{1}\geq H\left(X|U_{k-1}\right)$. Thus, there exists *Y* that satisfies $H\left(X|Y\right)=D_{1}$ and $U_{k}=Y{⊕}_{k}Z_{\epsilon }^{\left(k\right)}$ for some $0\leq \epsilon \leq \epsilon_{k}$. This implies that

$$\begin{array}{c} {\hat{X}}^{⋆}=Z_{\epsilon_{s}}^{\left(s\right)}{⊕}_{s}\left[Z_{\epsilon_{s-1}}^{\left(s-1\right)}{⊕}_{s-1}\cdots \left(Z_{\epsilon_{k+1}}^{\left(k+1\right)}{⊕}_{k+1}\left(Z_{\epsilon }^{\left(k\right)}{⊕}_{k}Y\right)\right)\right]. \end{array}$$

Similar to the previous section, we assume that $p_{X|Y}\left(\cdot |y_{1}\right)\neq p_{X|Y}\left(\cdot |y_{2}\right)$ if $y_{1}\neq y_{2}$. Then, we can set $Q=p_{X|Y}\left(\cdot |Y\right)$ which satisfies the conditions for successive refinability.

### 4.3. Iterative Lossy Compression Algorithm

The joint distribution from the previous section naturally suggests a simple successive refinement scheme. Consider the lossy compression problem where the source is i.i.d. $∼p_{X}$ and the distortion measure is $d:X\times \hat{X}\to \left[0,\infty \right)$. Let *D* be the target distortion, and R>R(D) be the rate of the scheme where R(D) is the rate-distortion function. Let $p_{X,{\hat{X}}^{⋆}}$ be the rate-distortion achieving distribution.

For block length *n*, we propose a new lossy compression scheme that mimics successive refinement with s−1 decoders. Similar to the previous section, let $\epsilon_{k}=\left(k-1\right)\min_{u}p_{U_{k}}\left(u\right)$ and

$$\begin{array}{cc} {\hat{X}}^{⋆}=U_{s}= & U_{s-1}{⊕}_{s}Z_{\epsilon_{s}}^{\left(s\right)}\\ U_{s-1}= & U_{s-2}{⊕}_{s-1}Z_{\epsilon_{s-1}}^{\left(s-1\right)}\\ \vdots \\ U_{2}= & U_{1}{⊕}_{2}Z_{\epsilon_{2}}^{\left(2\right)}. \end{array}$$

We further let $R_{k-1}>I\left(X;U_{k}\right)-I\left(X;U_{k-1}\right)$ for 2≤k≤s that satisfy $R=\sum_{k=2}^{s}R_{k-1}$. Now, we are ready to describe our coding scheme. Generate a sub-codebook $C_{1}=\left\{z^{n}\left(1,m\right):1\leq m\leq e^{R_{1}}\right\}$ where each sequence is generated according to $Z^{n}∼$ i.i.d. $p_{Z_{\epsilon_{2}}^{\left(2\right)}}$ for all *m*. Similarly, generate sub-codebooks $C_{k}=\left\{z^{n}\left(k,m\right):1\leq m\leq e^{nR_{k}}\right\}$ for 2≤k≤s−1 where each sequence is generated according to $Z^{n}∼$ i.i.d. $p_{Z_{\epsilon_{k+1}}^{\left(k+1\right)}}$ for all *m*.

Upon observing $x^{n}\in X^{n}$, the encoder finds $m_{1}\in C_{1}$ that minimizes $d_{1}\left(x^{n},z^{n}\left(1,m_{1}\right)\right)$ where the distortion measure $d_{1}\left(\cdot ,\cdot \right)$ is defined as follows.

$$\begin{array}{c} d_{1}\left(x^{n},z^{n}\right)=\frac{1}{n}\sum_{i=1}^{n}\log\frac{1}{p_{X|U_{2}}\left(x_{i}|z_{i}\right)}. \end{array}$$

Note that $d_{1}\left(x,z\right)$ is simply the logarithmic loss between *x* and $p_{X|U_{2}}\left(\cdot |z\right)$.

Similarly, for 2≤k≤s−1, the encoder iteratively finds $m_{k}\in C_{k}$ that minimizes $d_{k}\left(x^{n},\left[\left[z^{n}\left(1,m_{1}\right){⊕}_{3}\cdots {⊕}_{k}z^{n}\left(k-1,m_{k-1}\right)\right]{⊕}_{k+1}z^{n}\left(k,m_{k}\right)\right]\right)$ where

$$\begin{array}{c} d_{k}\left(x^{n},z^{n}\right)=\frac{1}{n}\sum_{i=1}^{n}\log\frac{1}{p_{X|U_{k+1}}\left(x_{i}|z_{i}\right)}. \end{array}$$

Upon receiving $m_{1},m_{2},\ldots ,m_{s-1}$, the decoder reconstructs

$$\begin{array}{c} {\hat{x}}^{n}=\left[\left[z^{n}\left(1,m_{1}\right){⊕}_{3}z^{n}\left(2,m_{2}\right)\right]⊕\cdots {⊕}_{s}z^{n}\left(s-1,m_{s-1}\right)\right]. \end{array}$$

Suppose $R_{1}\approx R_{2}\approx \cdots \approx R_{s-1}\approx \frac{R}{s-1}$, and L=s−1. Similar to [12,14], this scheme has two main advantages compare to naive random coding scheme. First, the number of codewords in the proposed scheme is $L\cdot e^{nR/L}$, while the naive scheme requires $e^{nR}$ codewords. Also, in each iteration, the encoder finds the best codeword among $e^{nR/L}$ sub-codewords. Thus, the overall complexity is $L\cdot e^{nR/L}$ as well. On the other hand, the naive scheme requires $e^{nR}$ complexity.

**Remark** **3.** *The proposed scheme constructs ${\hat{X}}^{n}$ from binary sequences. The reconstruction after each stage can be viewed as*

$$\begin{array}{c} u_{k}^{n}\left(m_{1},\ldots m_{k-1}\right)=\left[\left[z^{n}\left(1,m_{1}\right){⊕}_{3}\cdots \right]{⊕}_{k}z^{n}\left(k-1,m_{k-1}\right)\right] \end{array}$$

*where $0\leq u_{k}\leq k-1$. Thus, the decoder starts from binary sequence $u_{2}^{n}\left(m_{1}\right)$, and the alphabet size increases by 1 at each iteration. After (s−1)-th iteration, it reaches the final reconstruction ${\hat{X}}^{n}$ where the size of alphabet is s.*

## 5. Conclusions

To conclude our discussion, we summarize our main contributions. In the context of successive refinement problem, we showed another universal property of logarithmic loss that any discrete memoryless source is successively refinable as long as the intermediate decoders operate under logarithmic loss. We applied the result to the point-to-point lossy compression problem and proposed a lossy compression scheme with lower complexity.

## Appendix A. Proof of Lemma 2

For y≤s−1,

$$\begin{array}{cc} p_{X|Y}\left(x|y\right)= & \sum_{\hat{x}}p_{X|{\hat{X}}^{⋆}}\left(x|\hat{x}\right)p_{{\hat{X}}^{⋆}|Y}\left(\hat{x}|y\right)\\ = & p_{X|{\hat{X}}^{⋆}}\left(x|y\right). \end{array}$$

On the other hand, for y=s,

$$\begin{array}{cc} p_{X|Y}\left(x|y\right)= & \sum_{\hat{x}}p_{X|{\hat{X}}^{⋆}}\left(x|\hat{x}\right)p_{{\hat{X}}^{⋆}|Y}\left(\hat{x}|y\right)\\ = & \sum_{\hat{x}}p_{X|{\hat{X}}^{⋆}}\left(x|\hat{x}\right)p_{{\hat{X}}^{⋆}}\left(\hat{x}\right)\\ = & p_{X}\left(x\right). \end{array}$$

Let $j_{X}\left(x,D\right)$ be *d*-tilted information [15]:

$$\begin{array}{c} j_{X}\left(x,D\right)=\log\frac{1}{E\left[\exp\{\lambda^{⋆}D-\lambda^{⋆}d\left(x,{\hat{X}}^{⋆}\right)\}\right]}. \end{array}$$

where $\lambda^{⋆}=-R^{\prime }\left(D\right)$ and the expectation is with respect to marginal distribution $p_{{\hat{X}}^{⋆}}$. Csiszár [16] showed that for $p_{{\hat{X}}^{⋆}}$-almost every $\hat{x}$,

$$\begin{array}{cc} j_{X}\left(x,D\right)= & \log\frac{p_{X,{\hat{X}}^{⋆}}\left(x,\hat{x}\right)}{p_{X}\left(x\right)p_{{\hat{X}}^{⋆}}\left(\hat{x}\right)}+\lambda^{⋆}d\left(x,\hat{x}\right)-\lambda^{⋆}D\\ = & \log\frac{p_{X|{\hat{X}}^{⋆}}\left(x|\hat{x}\right)}{p_{X}\left(x\right)}+\lambda^{⋆}d\left(x,\hat{x}\right)-\lambda^{⋆}D. \end{array}$$

If $p_{X|{\hat{X}}^{⋆}}\left(x|{\hat{x}}_{1}\right)=p_{X|{\hat{X}}^{⋆}}\left(x|{\hat{x}}_{2}\right)$ for all *x*, it implies that $d\left(x,{\hat{x}}_{1}\right)=d\left(x,{\hat{x}}_{2}\right)$ for all *x* which contradicts our assumption. On the other hand, if $p_{X|{\hat{X}}^{⋆}}\left(x|{\hat{x}}_{1}\right)=p_{X}\left(x\right)$ for all *x*, it also contradicts our assumption. Thus, $p_{X|Y}\left(\cdot |y\right)$ are different from each other for all 0≤y≤s.

## Appendix B. Proof of the Special Case of Theorem 2

Similar to the main proof of Theorem 2, we assume $\hat{X}=Y=\left\{0,1,\ldots ,s-1\right\}$. Suppose there exists $\hat{x}$ such that $p_{X|{\hat{X}}^{⋆}}\left(x|\hat{x}\right)=p_{X}\left(x\right)$ for all *x*. Without loss of generality, we assume $\hat{x}=0$, i.e., $p_{X|{\hat{X}}^{⋆}}\left(x|0\right)=p_{X}\left(x\right)$ for all *x*.

Consider a random variable Y∈Y with the following conditional pmf for some 0≤ϵ≤1:

$$\begin{array}{c} p_{Y|{\hat{X}}^{⋆}}\left(y|\hat{x}\right)=\left\{\begin{array}{cc} 1 & \mathrm{if}\;\hat{x}=y=0\\ \epsilon & \mathrm{if}\;\hat{x}\neq 0\;\mathrm{and}\;y=0\\ 1-\epsilon & \mathrm{if}\;\hat{x}=y\neq 0.\\ 0 & \mathrm{otherwise}. \end{array}\right. \end{array}$$

It is clear that $H\left(X|Y\right)=H\left(X|{\hat{X}}^{⋆}\right)$ if ϵ=0 and H(X|Y)=H(X) if ϵ=1. Since $H\left(X|{\hat{X}}^{⋆}\right)\leq D_{1}$, there exists an 0≤ϵ≤1 such that $H\left(X|Y\right)=D_{1}$. We also have $Q=p_{X|Y}\left(\cdot |Y\right)$ and $q^{\left(y\right)}=p_{X|Y}\left(\cdot |y\right)$ for all y∈Y. The following lemma implies the one-to-one mapping between *q* and *y*.

**Lemma** **A1.**
*If $p_{X|Y}\left(x|y_{1}\right)=p_{X|Y}\left(x|y_{2}\right)$ for all x∈X, then $y_{1}=y_{2}$.*

**Proof.** If y=0, the conditional distribution $p_{{\hat{X}}^{⋆}|Y}\left(\hat{x}|y\right)$ is given by

$$\begin{array}{c} p_{{\hat{X}}^{⋆}|Y}\left(\hat{x}|y\right)=\left\{\begin{array}{cc} \frac{p_{{\hat{X}}^{⋆}}\left(0\right)}{\left(1-\epsilon \right)p_{{\hat{X}}^{⋆}}\left(0\right)+\epsilon } & \mathrm{if}\;\hat{x}=0\\ \frac{\epsilon \cdot p_{{\hat{X}}^{⋆}}\left(\hat{x}\right)}{\left(1-\epsilon \right)p_{{\hat{X}}^{⋆}}\left(0\right)+\epsilon } & \mathrm{if}\;\hat{x}\neq 0. \end{array}\right. \end{array}$$

Then,

$$\begin{array}{cc} p_{X|Y}\left(x|y\right)= & \sum_{\hat{x}}p_{X|{\hat{X}}^{⋆}}\left(x|\hat{x}\right)p_{{\hat{X}}^{⋆}|Y}\left(\hat{x}|0\right)\\ = & p_{X|{\hat{X}}^{⋆}}\left(x|0\right)\frac{p_{{\hat{X}}^{⋆}}\left(0\right)}{\left(1-\epsilon \right)p_{{\hat{X}}^{⋆}}\left(0\right)+\epsilon }+\sum_{\hat{x}\neq 0}p_{X|{\hat{X}}^{⋆}}\left(x|\hat{x}\right)\frac{\epsilon \cdot p_{{\hat{X}}^{⋆}}\left(\hat{x}\right)}{\left(1-\epsilon \right)p_{{\hat{X}}^{⋆}}\left(0\right)+\epsilon }\\ = & p_{X|{\hat{X}}^{⋆}}\left(x|0\right)\frac{\left(1-\epsilon \right)\cdot p_{{\hat{X}}^{⋆}}\left(0\right)}{\left(1-\epsilon \right)p_{{\hat{X}}^{⋆}}\left(0\right)+\epsilon }+p_{X}\left(x\right)\frac{\epsilon }{\left(1-\epsilon \right)p_{{\hat{X}}^{⋆}}\left(0\right)+\epsilon }\\ = & p_{X|{\hat{X}}^{⋆}}\left(x|0\right) \end{array}$$

where the last equality is because $p_{X|{\hat{X}}^{⋆}}\left(x|0\right)=p_{X}\left(x\right)$ for all *x*. In other words, $p_{X|Y}\left(x|0\right)=p_{X|{\hat{X}}^{⋆}}\left(x|0\right)$. On the other hand, if y≠0, the conditional distribution $p_{{\hat{X}}^{⋆}|Y}\left(\hat{x}|y\right)$ is given by

$$\begin{array}{c} p_{{\hat{X}}^{⋆}|Y}\left(\hat{x}|y\right)=\left\{\begin{array}{cc} 1 & \mathrm{if}\;\hat{x}=y\\ 0 & \mathrm{otherwise}. \end{array}\right. \end{array}$$

Then,

$$\begin{array}{cc} p_{X|Y}\left(x|y\right)= & \sum_{\hat{x}}p_{X|{\hat{X}}^{⋆}}\left(x|\hat{x}\right)p_{{\hat{X}}^{⋆}|Y}\left(\hat{x}|y\right)\\ = & p_{X|{\hat{X}}^{⋆}}\left(x|y\right). \end{array}$$

As we have seen in Appendix A, $p_{X|{\hat{X}}^{⋆}}\left(\cdot |{\hat{x}}_{1}\right)$ cannot be equal to $p_{X|{\hat{X}}^{⋆}}\left(\cdot |{\hat{x}}_{2}\right)$ if ${\hat{x}}_{1}\neq {\hat{x}}_{2}$. Since $p_{X|Y}\left(x|y\right)=p_{X|{\hat{X}}^{⋆}}\left(x|y\right)$ for all *x*, we can say that $p_{X|Y}\left(x|y_{1}\right)=p_{X|Y}\left(x|y_{2}\right)$ for all *x* implies $y_{1}=y_{2}$. □

The remaining part of the proof is exactly the same as the main proof.
